# Plasticity of Daily Behavioral Rhythms in Foragers and Nurses of the Ant *Camponotus rufipes*: Influence of Social Context and Feeding Times

**DOI:** 10.1371/journal.pone.0169244

**Published:** 2017-01-18

**Authors:** Stephanie Mildner, Flavio Roces

**Affiliations:** Department of Behavioral Biology and Sociobiology (Zoology II), Biocenter, University of Würzburg, Würzburg, Germany; University of Texas Southwestern Medical Center, UNITED STATES

## Abstract

Daily activities within an ant colony need precise temporal organization, and an endogenous clock appears to be essential for such timing processes. A clock drives locomotor rhythms in isolated workers in a number of ant species, but its involvement in activities displayed in the social context is unknown. We compared locomotor rhythms in isolated individuals and behavioral rhythms in the social context of workers of the ant *Camponotus rufipes*. Both forager and nurse workers exhibited circadian rhythms in locomotor activity under constant conditions, indicating the involvement of an endogenous clock. Activity was mostly nocturnal and synchronized with the 12:12h light-dark-cycle. To evaluate whether rhythmicity was maintained in the social context and could be synchronized with non-photic zeitgebers such as feeding times, daily behavioral activities of single workers inside and outside the nest were quantified continuously over 24 hours in 1656 hours of video recordings. Food availability was limited to a short time window either at day or at night, thus mimicking natural conditions of temporally restricted food access. Most foragers showed circadian foraging behavior synchronized with food availability, either at day or nighttime. When isolated thereafter in single locomotor activity monitors, foragers mainly displayed arrhythmicity. Here, high mortality suggested potential stressful effects of the former restriction of food availability. In contrast, nurse workers showed high overall activity levels in the social context and performed their tasks all around the clock with no circadian pattern, likely to meet the needs of the brood. In isolation, the same individuals exhibited in turn strong rhythmic activity and nocturnality. Thus, endogenous activity rhythms were inhibited in the social context, and timing of daily behaviors was flexibly adapted to cope with task demands. As a similar socially-mediated plasticity in circadian rhythms was already shown in honey bees, the temporal organization in *C*. *rufipes* and honey bees appear to share similar basic features.

## Introduction

Ant societies are well known for their decentralized structure and organization of activities through division of labor. Colony tasks are usually performed simultaneously inside and outside the nest by different worker groups. In general, task allocation in ants is based on both temporal and size polyethism [1]. In the nectar-feeding ant *Camponotus rufipes*, for instance, media-sized workers engage in tasks both inside and outside the nest [2]. Further task allocation is based on worker age. Young workers stay inside the nest engaging in nursing, whereas older workers leave the nest to forage [3]. Beyond the observed plasticity in task allocation in social insects [4, 5], daily activities should be synchronized among individuals inside and outside the nest to generate a coordinated colony response, especially under varying abiotic and biotic environmental factors.

### Activity rhythms outside the nest

It is an open question the extent to which workers engaged in different nest tasks coordinate their activities within an ant colony. While some tasks like nest digging or brood care do not appear at first glance to underlie daily changes or to be synchronized with biotic or abiotic factors, other tasks such as foraging and feeding of brood are expected to be tightly synchronized in order to increase colony efficiency. In nectar-feeding ants of the genus *Camponotus*, which lack food stores inside their nests, workers repeatedly visit renewable nectar resources such as extrafloral nectaries or aphid colonies, and their foraging activity is expected to be synchronized with the availability of carbohydrate sources. As a consequence, even prey collection may be limited to a certain phase of the day. The access to food sources is likely to be temporally restricted by daily cycles in nectar production [6, 7, 8], changing environmental conditions [9–13] or competitors [14, 15]. As such factors may have synergistic effects [16, 17], foraging activity needs to be scheduled to the right time of the day within a species-specific temporal niche. Studies on other nectar-feeding ant species focusing on the finding of food at a certain time of the day already provided further evidence for a robust time sense in these ants [18–23]. And in fact, *C*. *rufipes* workers have the ability to measure short time intervals while visiting nectar sources and to adjust their visiting times at food patches accordingly [24]. Although workers from this species predominantly forage during the nighttime [2], reports about occasional diurnal foraging [25–27] highlight the flexible timing of activities. Activity shifts might represent a seasonal adaptation to changing environmental factors or food availability. Since such foraging rhythms were so far only demonstrated at the colony level, the questions whether colony-wide foraging rhythms reflect the rhythmical activity of single workers and are modulated by the temporal availability of food remained open. Therefore we investigated behavioral activity patterns of individual foragers under different feeding schedules.

### Activity rhythms within the nest

There is also evidence that coordinated activity rhythms occur among workers inside an ant nest [28, 29], which may reduce redundancy and therefore increase the efficiency of collective behaviors such as nest building and brood care. Efficient brood care seems to be of high importance because it is linked to colony growth and survival. Groups of *Leptothorax* ants, for instance, showed synchronized peaks of locomotor activity inside the nest [30, 31], with synchrony increasing with group size and presence of brood [32]. It was hypothesized that synchronized locomotion resembles synchronized brood care [28, 29]. These studies, however, quantified only locomotor activity inside the nest for short time intervals, so that the question whether specific nursing behaviors undergo a daily cycle remained unexplored. Studies focusing on brood relocation for temperature control in the ant *Camponotus mus*, however, provided unequivocal evidence about the occurrence of endogenous, self-sustained bimodal rhythms in thermopreference for relocation of the immobile brood inside the nest by nurse workers [33–36]. Rhythmic brood translocation of pupae optimizes brain development and could affect sensory processing and learning abilities in adult ants [37], being therefore relevant for colony functioning. To what extent other nursing activities such as feeding of brood follow a daily rhythm, for instance locked-on to daily rhythms in food collection by foragers, is still unknown. Therefore, we quantified daily activity patterns in nurses within the nest as compared to the activity patterns of foragers.

### Activity rhythms and endogenous clocks

Almost all organisms have an endogenous clock that enables them to schedule daily activities. Core element of such clock systems is an endogenous oscillator, which generates multiple rhythms in metabolism and behavior. Under suitable, predictable daily changes in the environment (′zeitgeber′, e.g. the light-dark cycle), the oscillator generates entrained rhythms with periodicities of 24 hours. Under the absence of zeitgebers, rhythms keep free running with periodicities close to 24 hours. In several ant species, single isolated workers exhibit strong circadian rhythms in locomotor activity, thus providing evidence about the existence of an endogenous clock [38–40]. As shown for different ant species, worker castes differ in their locomotor activity patterns. In *Pogonomyrmex* and *Camponotus* ants, foragers exhibited rhythmic activity patterns, whereas nest workers showed no marked activity peaks [41, 42]. Conversely, nurse workers in *Diacamma sp*. Showed strong circadian rhythms, and foragers were arrhythmic with much higher activity levels [43]. In both cases, locomotor rhythms are hypothesized to reflect the activity profiles of the castes within the colony. But as rhythms of specific behaviors of the castes within the nest have not been analyzed so far, those locomotor rhythms can not be linked to true behavioral rhythms in the social context. In a broader sense, we lack information about the extent to which endogenous rhythms are involved in the scheduling of daily behaviors in the social context and in the synchronization among workers performing different tasks in ants. In honey bees, nurses perform their work inside the hive around the clock, with no apparent rhythmicity [44–46]. In contrast, foraging bees exhibit robust diurnal patterns of activity in the social context, strongly linked to food availability [47–50]. As nurses show rhythmic locomotor activity like foragers when isolated from the colony, their endogenous rhythmicity is suppressed in the social context [51]. Therefore, plasticity in daily activity rhythms is linked to division of labor and strongly depends on the social context [52]. To evaluate the impact of social environment on daily rhythms in bees, locomotor activity of isolated bees has been recorded under contact to microclimate (including pheromones) of a small colony [53]. Workers synchronized their activity with the activity of the hive and more workers exhibited rhythmic locomotor patterns than isolated bees without any contact, which emphasizes the modulating effects of the social environment. It is unknown whether socially-mediated plasticity in activity rhythms represents a conserved, adaptive trait in other social insects like ants as well. To provide evidence of socially-mediated plasticity in activity rhythms in ants, we compared the locomotor activity rhythms in both isolated forager and nurse workers with their behavioral rhythms in the social context of the colony.

### Aim of the study

The present study was aimed at investigating the link between division of labor and temporal organization in the nectar-feeding ant *C*. *rufipes*. We studied activity patterns of foragers, as representatives for outside-nest activities, and nurses, which perform inside-nest behaviors. Both castes are closely linked through food availability, as foragers are responsible for food collection and nurses subsequently feed the brood with the collected food. Daily rhythms of locomotor activity in isolated workers of both castes were quantified under controlled conditions in a first experiment, to provide evidence about the presence of an endogenous clock. To highlight possible effects of the social context on activity rhythms, we monitored daily activity of individually-identified foragers and nurses in small colonies in a second experiment, and compared the patterns with those observed in isolation. As temporal changes in food availability may strongly influence daily activities, we presented colonies in different series with daily pulses of food availability as well as *ad libitum* feeding regimes. Therefore, we analyzed the potential link between temporal changes in resource availability, timing of foraging behavior, and synchronization with inside-nest nursing behavior in the ant *C*. *rufipes*.

## Materials and Methods

### Study system

*C*. *rufipes* is a nectar-collecting ant species distributed over broad ranges in South America. For the laboratory studies, queens were collected during their mating flights in December 2011 and 2014 in La Coronilla, Uruguay (33°53'25.2"S, 53°31'27.6"W) and were brought to the laboratory at the University of Würzburg, Germany. The species *C*. *rufipes* is not endangered nor protected. Export permits were issued by the Departamento de Fauna de la Dirección General de Recursos Naturales Renovables, Ministerio de Ganadería, Agricultura y Pesca, Uruguay. Colonies were raised in plaster nests under constant conditions (25°C, 50%rH) and a 12:12h LD-cycle (300 lux during the light phase). Queenright colonies consisted of several thousand workers and were fed *ad libitum* with water, diluted honey and pieces of cockroaches. This study complies with the ethical guidelines of the country where the research was carried out.

### Experiment 1: Locomotor rhythms of isolated foragers and nurses of the ant *C*. *rufipes*

To study daily patterns in locomotion and their endogenous nature in nurses and foragers in *C*. *rufipes*, we employed a commercially available locomotor activity recording system (LAM 16; TriKinetics, Waltham, USA). This recording method is well established for *Drosophila* [54, 55] and was recently adapted to monitor social insects like bees and wasps [53, 56]. One monitor (33 x 12 x 20 cm) allows the simultaneous recording of 32 individuals in an automated way for infinite time. For that, insects are placed individually into glass tubes (length: 10 cm, diameter: 1.5 cm), where their locomotor activity is monitored by three infrared light beams in the center of each glass tube. Automatic counts of the interruptions of the light beams caused by the insect’s locomotor activity accumulate for each individual in self-selected time windows (in our experiments in ten minute bins) and are then communicated to a computer equipped with the corresponding recording program. Raw data can be processed later on and be used for example to display activity rhythms in form of actograms (e.g. via ActogramJ, [57]).

**Fig 1:**
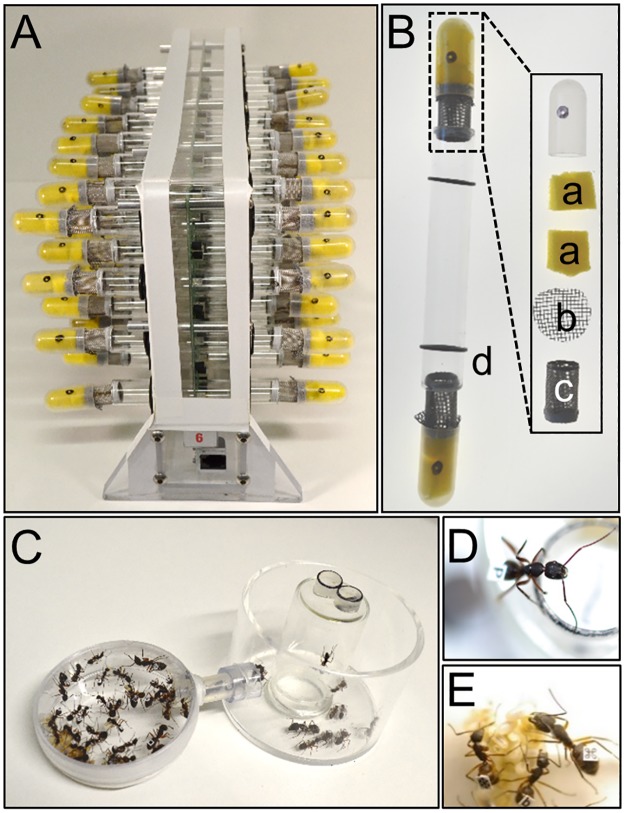
Experimental setups for monitoring both locomotor and behavioral activities. (A): Modified locomotor activity monitors (TriKinetics). Activity of single individuals was recorded in isolation from the social context via crossings of infrared light beams in the center of the glass tubes (B). *Ad libitum* supply of 5% (w/w) sugar water was provided through sponges (a), separated from the glass tube by plastic nets (b) to allow fluid intake while avoiding chewing by the ant. A metal mesh tunnel segment (c) connected the glass tubes with the feeding devices to provide aeration. Glass tubes were fixed to the monitors by o-rings (d). (C) Experimental setup for the determination of caste affiliation (experiment 1) and for the analysis of daily activities in the social context (experiment 2). Subcolony of individually marked workers of *C*. *rufipes* in transparent setup with enclosed brood chamber (left) and open foraging arena with feeding platform (right). (D): forager on the feeding platform. (E): nurses at the brood pile.

The monitors were modified in our study to enable the recording of isolated ants over weeks (Fig 1A and 1B). As ants are prone to desiccation in isolation of the colony and need, besides nutrition, constant access to water, we supplied them with *ad libitum* access to a low concentrated sugar solution. We designed special feeding caps that sealed the glass tubes at both ends (Fig 1B), so the insects would not stay at one end of the glass tube and display reduced overall activity. Plastic caps were filled with sponges soaked with 5% sugar water (w/w; 1.5 ml volume per cap) and separated from the insects with plastic nets to keep them within the recording section of the tube. The sugar water needed to be refilled every third day, which was accomplished through injections in a single perforation of the plastic cap at random time points without interfering the recordings. A mesh tunnel connected the feeding caps with the glass tubes to provide aeration.

To record locomotor activity of the two different worker castes, we initially built three “subcolonies” from three *C*. *rufipes* queenright colonies (named colony H, Q and 14). A subcolony was a queenless group of several workers and brood settled in an artificial brood chamber (diameter: 5.5 cm, height: 1.5 cm) with access to an outside foraging arena (diameter: 6.3 cm, height: 4.6 cm) via one tunnel (diameter: 0.6 cm, length: 3.5 cm), thus representing a functional group of workers that displayed both foraging and nursing behaviors. There is no evidence that the absence of the queen affects caste affiliation. It may reduce the total activity in both castes as compared to that of natural colonies, thus leading to an underestimation of activities in our experiments [58, 59]. Subcolonies of 50 workers and 120 larvae were held under constant conditions (25°C, 50%rH) and a 12:12h LD-cycle (3000 lux during light phase) in transparent acrylic glass nest setups (Fig 1C) placed in incubators (I-30BLL, CLF PlantClimatics GmbH). In this way it was assured that all workers were exposed to the LD-cycle. To build the subcolonies and promote division of labor, we collected ants with varying body sizes both from the brood chamber as well as the foraging areas of the large queenright colonies. An *ad libitum* supply of food (honey-water, chopped cockroaches and water) was provided daily on an elevated platform (diameter: 2.6 cm, height: 5.0 cm) in the foraging arena. The use of the platform assured that foragers actively searched for food, allowing their distinction from other outside workers like guards. All workers were marked individually with printed paper tags glued to their gaster with acrylic paint (KÜNSTLER.FARBEN.FABRIK, Hallerndorf, Germany; Fig 1D and 1E), thus affiliation of individuals to the forager or to the nurse caste could be determined based on their behavior and location. Foragers were considered as those ants present at the foraging platform collecting food, while nurses were those workers tending brood inside the brood chamber. Caste affiliation was determined visually on a daily basis throughout one week after the subcolonies were established, which simultaneously represented the 1-week-entrainment phase under the LD-cycle.

The small subcolonies allowed unequivocal recognition of tagged individuals, especially inside the brood chamber, but as a consequence of the reduced subcolony size, only few workers engaged as foragers. To obtain adequate sample sizes for locomotor activity monitoring of foragers, we additionally built larger subcolonies with 150 workers and greater numbers of brood items in a larger setup (brood chamber: 9x9x5.5 cm, foraging arena: 19x19x9 cm, feeding platform: diameter: 8.5 cm, height: 3.5 cm). Workers collecting food at the platform were considered as foragers and were marked daily throughout one week.

Thereafter, subcolonies were discontinued and single identified individuals of known caste were placed in the locomotor activity monitors and recorded over three weeks under different light regimes, each lasting one week. After one day of settling in, daily activity of single ants was recorded for seven days under the similar 12:12h LD-cycle used for the subcolonies. In the second week of recording, the LD-cycle was delayed for six hours, to test if individuals were able to resynchronize their activity after the shift. Finally, locomotor activity was recorded under constant darkness to provide evidence of endogenous activity patterns and to quantify free-running periods.

Activity measured over the whole recording period (22 days) was displayed in form of actograms via ActogramJ for all individuals. For the recording periods under the LD-cycle as well as the phase-shifted LD-cycle, we generated average activity patterns of all surviving ants based on seven days of each observation period (LD-cycle: n_Foragers_ = 78, n_Nurses_ = 46; phase-shifted LD-cycle: n_Forager_ = 61, n_Nurses_ = 37). Calculations of total activity levels as well as of the relative nocturnal activity (normalized for the total activity of every individual) on the second day of the respective recording period were selected to avoid pseudoreplications, and so to quantitatively compare activity patterns between the castes under the three light regimes (Mann-Whitney U-tests). For further description of the ants’ endogenous clock we calculated the period values of the individual activity under the LD-cycles and constant darkness by periodogram analysis (Lomb-Scargle-method, significance level α = 0.5; ActogramJ), and tested for differences between the castes (Mann-Whitney U-tests). The effects of caste on survival rate and rhythmicity were analyzed by χ^2^ tests. All statistical analysis were performed in STATISTICA (StatSoft, Inc., Version 10.0) after testing data sets for normal distribution via Shapiro-Wilks test.

### Experiment 2: Caste-dependent plasticity in daily behavioral rhythms

#### Daily behavioral activity patterns of foragers and nurses in the social context

In order to investigate caste-specific daily activities, we built 32 subcolonies of individually marked ants as described for Experiment 1 (Fig 1C, see previous paragraph for details) from four *C*. *rufipes* queenright colonies (named colony J, O, H and R).

To determine the synchronization abilities of workers with pulses of food availability, we carried out three experimental series with either time-restricted or *ad libitum* feeding regimes. In two series, food availability was restricted to a short time window either four hours after lights-on (**Z**eitgeber **T**ime: ZT 4; “daytime feeding”) or four hours after lights-off (ZT 16; “nighttime feeding”). A supply of 10% sugar water and ten freshly killed *Drosophila* flies proved to be sufficient for subcolony maintenance. Depending on the collecting rate of the ants, food was present in the foraging arena only for up to two hours. In the third series, which served as a control, subcolonies were fed *ad libitum* throughout the day to identify endogenous foraging times. Here, pieces of cockroaches were fed instead of *Drosophila* flies to ensure permanent food availability. The amount of sugar water and cockroaches exceeded the subcolony’s daily needs and were replaced daily at different times to avoid temporal cues.

Caste affiliation was determined on a daily basis throughout the first week after setting-up of the subcolony, which served as settling-in period and entrainment phase. In order to quantify all daily behaviors of the castes in a continuous way, we conducted 24-hour video recordings of the setups in the following week, i.e., after subcolonies had experienced the corresponding feeding regime for more than 7 but less than 14 days. Infrared cameras (CCD & CMOS, Conrad Electronic SE) and time-lapse analog video recorders (72-hour time-mode, Panasonic AG-6720/AG-6730) were used. Thereafter, an observer watched the videos and built ethograms for caste- and non-caste specific activities (Table 1) of randomly selected members of the two castes combined with their spatial location (TheObserver, Version 2.01, Noldus Information Technology). From this raw data, we calculated differences in total levels of inactivity per day between the castes for every feeding regime (Mann-Whitney U tests), as well as within castes over the feeding regimes (Kruskal-Wallis tests) under Bonferroni correction (α = 0.017). Average time budgets for all behavioral activities were quantified. Daily patterns of caste-specific activity were calculated in addition by summing all activities belonging to this category. To enable data visualization, activities were pooled in 10 minute bins and therefore displayed as proportion per bin. We calculated period values (τ) of behavioral rhythms of both castes under the three feeding regimes using the cosinor method (significance level: α = 0.05; Cosinor program, Refinetti, version 3.1). Period values were categorized as ultradian (<20h), circadian (24±4h) or infradian (>28h). Foraging activity for every subcolony was quantified as the relative number of ants present in the foraging arena at every half an hour of the video recording, as well as total foraging activity per 12h-dark or light phase. Foraging activity was compared between the two 12h-light and dark phases within feeding regimes (Wilcoxon signed-rank test) and between feeding regimes (Kruskal-Wallis test) under Bonferroni correction (α = 0.017).

**Table 1:** Activities displayed by single workers in the foraging arena (foragers) and in the brood chamber (nurses), monitored and continuously quantified over 24 h using time-lapse videos. Antennating: antennating objects, brood or conspecifics whilst standing. Walking: locomotor movement. Allo(grooming): self-grooming or grooming of conspecifics. Waste management: manipulating or carrying waste material (e.g. food remains, corpses). Trophallaxis: exchange of liquid food between workers. Food consumption: intake of food (foragers: ingestion of sugar water, water and feeding on *Drosophila* flies in the foraging arena; nurses: feeding on *Drosophila* flies in the brood chamber). Food transport: carrying *Drosophila*. Brood relocation: picking up and transporting larvae between the mandibles. Brood care: feeding liquid food to larvae or licking larvae.

Non-specific activities	Caste-specific activities	
	Foragers	Nurses
Antennating Walking (Allo)Grooming Waste management	Trophallaxis Food consumption Food transport	Brood relocation Food consumption Brood care

#### Daily locomotor activity of foragers and nurses in isolation from the social context

In order to evaluate the effects of the social context on the observed behavioral rhythms of foragers and nurses in the subcolonies, locomotor rhythms of all individually-identified workers were monitored over 8 days in isolation immediately after removal from the social context. The subsequent monitoring of locomotor activity served to verify the existence of a functional endogenous clock in the individuals. Experiments were performed using the modified locomotor activity monitors described above placed in incubators under constant conditions (25°C, 50%rH; Incubator: Rumed 1200, Rubarth Apparate GmbH; Incubator: I-30BLL, CLF PlantClimatics GmbH). A 12:12h LD-cycle was applied (4000–6000 lux during the light phase) to characterize daily locomotor rhythms. Data analysis was performed by calculating actograms (ActogramJ) and determining proportions of rhythmic individuals in each caste by periodogram analysis (Lomb-Scargle method, significance level α = 0.05), as well as the survival rate of castes after eight days of observation. The effect of caste and feeding regimes on survival rate and rhythmicity was analyzed by χ^2^ tests under Bonferroni correction (α = 0.017). Representative actograms were chosen for every caste and feeding regime based on the most common activity patterns (rhythmic and arrhythmic).

## Results

The complete datasets can be found in S1 Table.

### Experiment 1: Locomotor rhythms of isolated foragers and nurses of *C*. *rufipes*

**Fig 2:**
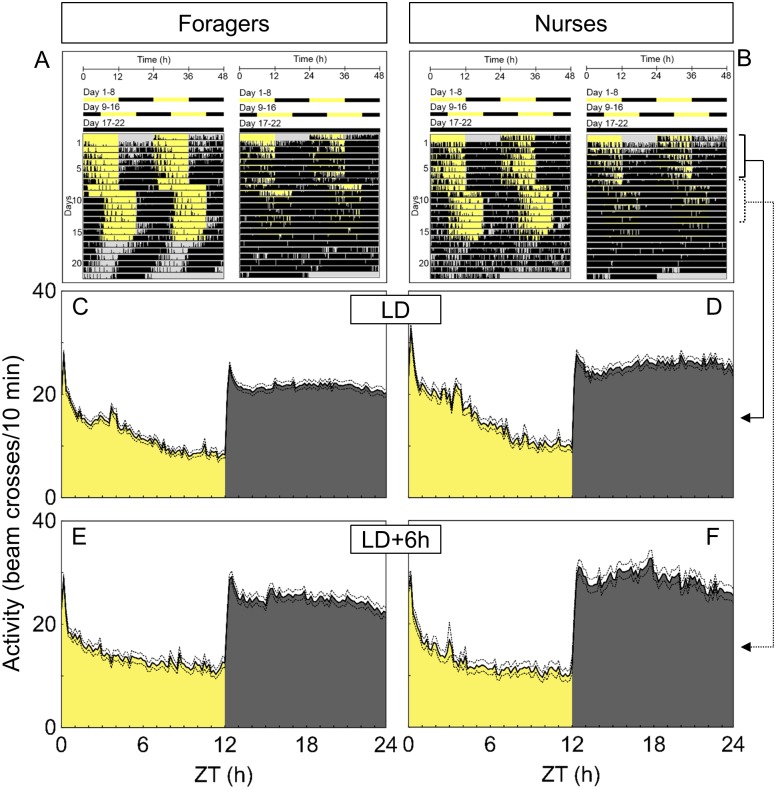
Locomotor activity rhythms of foragers and nurses in isolation from the social context. Top: Examples of actograms of single workers of each caste. Locomotor activity (indicated as black bars) is shown as double plot under a 12:12h LD-cycle (day 1–8), after a 6 hour phase delay of the LD-cycle (day 9–15) and constant darkness (day 16–22). (A): Actograms of one rhythmic (left) and one arrhythmic (right) forager; (B): Actograms of one rhythmic (left) and one arrhythmic (right) nurse. Bottom: Average activity (mean: solid lines; mean±SE: dashed lines) over 7 days under the respective light regime. (C): Foragers under a 12:12h LD-cycle (n = 78): (D): Nurses under a 12:12h LD-cycle (n = 46); (E): Foragers after a 6 hour delay of the LD-cycle (n = 61); (F): Nurses after a 6 hour delay of the LD-cycle (n = 37).

Although survival rates varied in both castes throughout the recordings, survival of nurses was only significantly lower than that of foragers during the last week of the recordings (Table 2; χ^2^ tests; LD: χ^2^ = 0.36, p = 0.5; phase-shifted LD: χ^2^ = 0.09, p = 0.8; DD: χ^2^ = 7.63, p = 0.006). There were no differences between foragers and nurses in rhythmicity under both LD-regimes as well as under constant darkness (Table 2, χ^2^ tests; LD: χ^2^ = 0.00, p = 0.9; phase-shifted LD: χ^2^ = 0.72, p = 0.4; DD: χ^2^ = 1.31, p = 0.2). Around 60% of individuals exhibited rhythmic activity patterns. In that case, foragers and nurses synchronized their activity rhythm strongly with the 12:12h LD-cycle (Fig 2A and 2B, actograms on the left, Day 1–8). The average activity patterns in both castes revealed lower activity levels during day times (Fig 2C and 2E) and sharp activity peaks after the two light transitions. After shifting the light regime by six hours, ants immediately resynchronized their activity (Fig 2A and 2B, actograms on the left, Day 9–16) and therefore displayed an almost identical average activity pattern as before the phase shift (Fig 2D and 2F). Total activity levels as well as relative levels in nocturnal activity were compared between castes to quantify differences in locomotor rhythms (Table 2). Nurses exhibited total activity levels higher than foragers, but only during the first recording phase under the LD-cycle (Mann-Whitney U-tests; LD: U(78,46) = 1203.5, z = -3.0, p = 0.002; phase-shifted LD: U(61,37) = 2060.0, z = -1.7, p = 0.09; DD: U(49,20) = 465.0, z = -0.3, p = 0.7). There were no significant differences in levels of nocturnality between the castes under both light regimes (Mann-Whitney U-tests; LD: U(78,46) = 1537.0, z = 1.3, p = 0.1; DD: U(61,37) = 1204.0, z = -0.8, p = 0.4). Here, foragers and nurses displayed similar period lengths close to 24 hours (Mann-Whitney U-tests; LD: U(74,45) = 1357.0, z = -1.7, p = 0.08; phase-shifted LD: U(60,36) = 1582.5, z = 1.2, p = 0.2). During constant darkness, locomotor activity rhythms of both castes drifted with similar endogenous periods shorter than 24 hours (foragers: 22.4±0.39 h, nurses: 23.1±0.76 h; Mann-Whitney U-test: U(44,18) = 1317.0, z = -1.1, p = 0.3).

**Table 2:** Survival rate, proportion of rhythmic individuals and activity characteristics of foragers and nurses isolated in locomotor activity monitors. Different letters show significant differences between castes within the three light regimes (significance level α = 0.05). Differences in survival and rhythmicity rates were evaluated via χ^2^ tests; differences in activity periods (τ), total activity levels and proportion of night activity were evaluated via Mann-Whitney-U tests.

		Survival (%)	Rhythmicity (%)	Period (h) [median±SE]	Total Activity (beam crosses/day) [median±SE]	Night activity (%) [median±SE]
**LD**	**Forager**	55.7^a^ (n = 78)	59.0^a^ (n = 46)	24.1±0.1^a^ (n = 74)	2040.5±139.2^a^ (n = 78)	68.1±1.6^a^ (n = 78)
	**Nurse**	80.7^a^ (n = 46)	58.7^a^ (n = 27)	24.0±0.12^a^ (n = 45)	2868.7±218.0^b^ (n = 46)	64.8±2.1^a^ (n = 46)
**LD+6h**	**Forager**	78.3^a^ (n = 61)	59.0^a^ (n = 36)	24.6±0.2^a^ (n = 60)	2616.1±229.3^a^ (n = 61)	69.8±1.8^a^ (n = 61)
	**Nurse**	64.9^a^ (n = 37)	65.8^a^ (n = 25)	23.9±0.3^a^ (n = 36)	3172.7±283.0^a^ (n = 37)	71.4±2.3^a^ (n = 37)
**DD**	**Forager**	80.3^a^ (n = 49)	55.1^a^ (n = 27)	22.4±0.39^a^ (n = 44)	2785.0±349.3^a^ (n = 49)	-
	**Nurse**	54.0^b^ (n = 20)	70.0^a^ (n = 14)	23.1±0.76^a^ (n = 18)	2634.0±621.8^a^ (n = 20)	-

### Experiment 2: Caste-dependent plasticity in daily behavioral rhythms

#### Daily behavioral activity patterns of foragers and nurses in the social context

**Fig 3:**
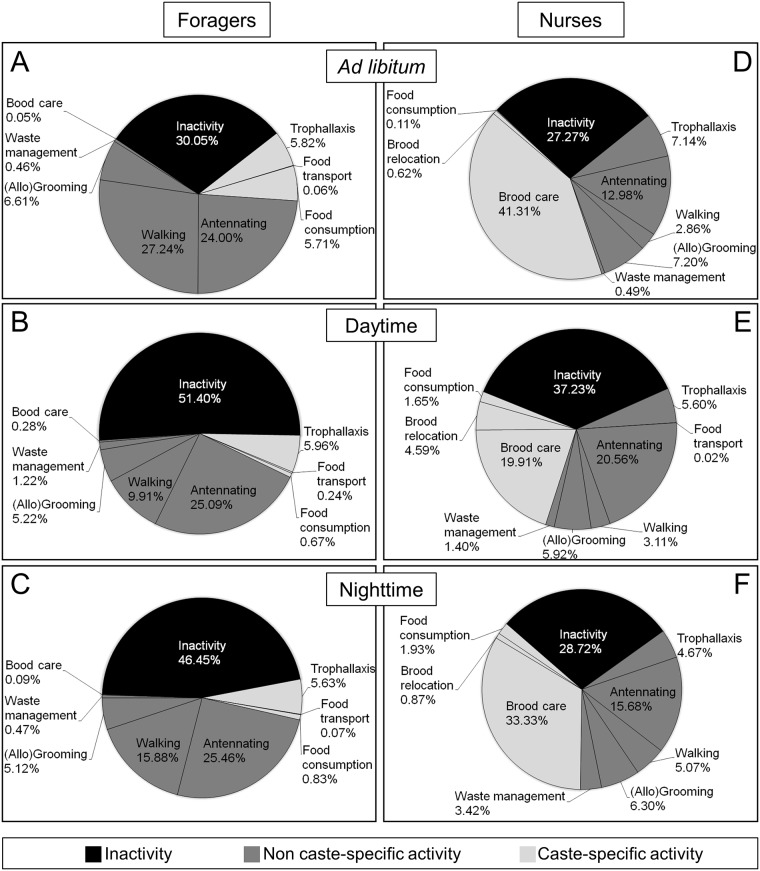
Daily time budgets for activities displayed in the social context for both foragers and nurses under the three feeding regimes. (A): foragers (n = 8) under *ad libitum* feeding; (B): foragers (n = 12) under daytime feeding; (C): foragers (n = 12) under nighttime feeding; (D): nurses (n = 13) under *ad libitum* feeding; (E): nurses (n = 12) under daytime feeding; (F): nurses (n = 12) under nighttime feeding. Black: proportion of inactivity, light grey: proportion of caste-specific activity, dark grey: proportion of non caste-specific activity.

With up to 50% of their time, foragers showed much higher levels of inactivity than nurses under the restricted feeding regimes (Fig 3; Mann-Whitney U-tests under Bonferroni correction, α = 0.017; daytime feeding: U(12,13) = 17.0, z = 3.3, p = 0.001; nighttime feeding: U(12,12) = 21.0, p = 0.003). Under *ad libitum* feeding, however, only foragers tended to be more active (Kruskal-Wallis tests under Bonferroni correction, α = 0.017; foragers: H(2, n = 32) = 4.23, p = 0.12; *ad libitum* vs. daytime: p = 0.13; *ad libitum* vs. nighttime: p = 0.42; daytime vs. nighttime: p = 1.0; nurses: H(2, n = 37) = 8.12, p = 0.017; *ad libitum* vs. daytime: p = 0.028; *ad libitum* vs. nighttime: p = 1.0; daytime vs. nighttime: p = 0.07) and therefore inactivity levels did not differ between castes (Mann-Whitney U-test, U(8,12) = 40.0, z = 0.58, p = 0.6). In addition, foragers had consistently lower total caste-specific activity levels than nurses in each of the feeding regimes (Fig 3; Mann-Whitney U-tests; *ad libitum* feeding: U(8,12) = 7.0, z = -3.12, p = 0.0018; daytime feeding: U(12,13) = 0.0, z = -4.2, p<0.001; nighttime feeding: U(12,12) = 0.0, z = -4.1, p<0.001). The daily time budgets of the castes showed that in foragers, antennating (~25%) and walking (10–27%) were the most dominant activities (Fig 3A, 3B and 3C). With 20 to 41% of time allocation, nurses spent their time predominantly with brood feeding (Fig 3D, 3E and 3F). Both castes engaged with 5 to 7% of their time in exchange and distribution of food via trophallaxis.

**Fig 4:**
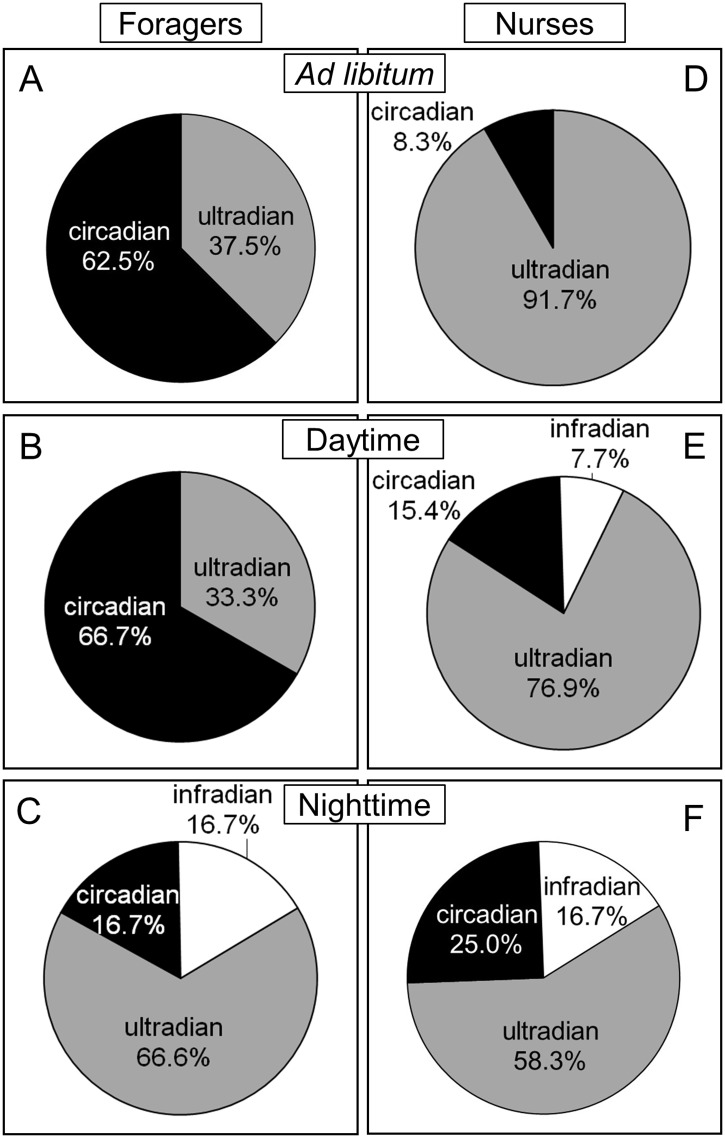
Percentage of foragers and nurses displaying either circadian, ultradian or infradian rhythms of caste-specific activities in the social context, under the three feeding regimes. Black: circadian rhythms (period lengths between 20 and 28h). Grey: ultradian rhythms (period lengths <20h). White: infradian rhythms (period lengths >28h) (A): foragers (n = 8) under *ad libitum* feeding; (B): foragers (n = 12) under daytime feeding; (C): foragers (n = 12) under nighttime feeding; (D): nurses (n = 13) under *ad libitum* feeding; (E): nurses (n = 12) under daytime feeding; (F): nurses (n = 12) under nighttime feeding.

**Fig 5:**
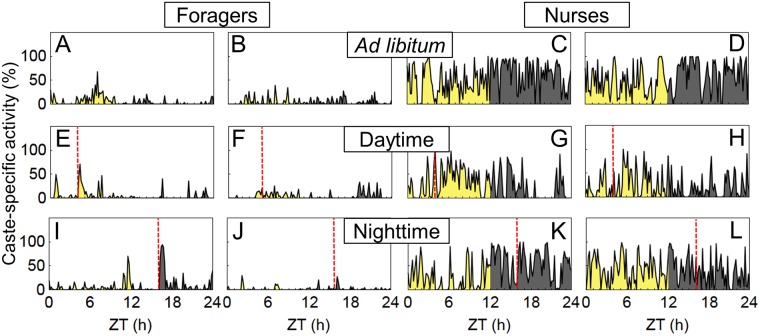
Examples of caste-specific activity patterns of both foragers and nurses in the social context, under the three feeding regimes. Activity of single workers is shown for the day phase (yellow area) and night phase (black area) in relation to zeitgeber time (ZT). Feeding times (daytime feeding: ZT 4; nighttime feeding: ZT 16) are indicated as dashed red lines. For both castes and the three feeding regimes, one circadian (left) and one ultradian (right) activity pattern are shown as examples. (A): Forager under *ad libitum* feeding, τ = 22.3h. (B): Forager under *ad libitum* feeding, τ = 4.5h. (C): Nurse under *ad libitum* feeding, τ = 21.3h. (D): Nurse under *ad libitum* feeding, τ = 2.4h. (E): Forager under daytime feeding, τ = 24.6. (F): Forager under daytime feeding, τ = 13.4h. (G): Nurse under daytime feeding, τ = 24.1h. (H): Nurse under daytime feeding, τ = 3.1h. (I): Forager under nighttime feeding, τ = 22.1. (J): Forager under nighttime feeding, τ = 2.8. (K): Nurse under nighttime feeding, τ = 22.4h. (L): Nurse under nighttime feeding, τ = 3.6h.

To characterize the rhythmicity of the behavioral patterns quantified in the social context, we calculated period values (τ) and summarized the percentage of foragers and nurses displaying either circadian, ultradian or infradian rhythms in their caste-specific activities (Fig 4). Under *ad libitum* and daytime feeding, more than 60% of foragers displayed circadian activity patterns (Fig 4A and 4B). Ultradian activity rhythms were detected in this caste under the three feeding regimes, but mostly under nighttime feeding (Fig 4C). For all feeding regimes, the majority of nurses displayed ultradian activity rhythms (Fig 4). Examples for both ultradian and circadian activity rhythms in the two castes are presented in Fig 5.

**Fig 6:**
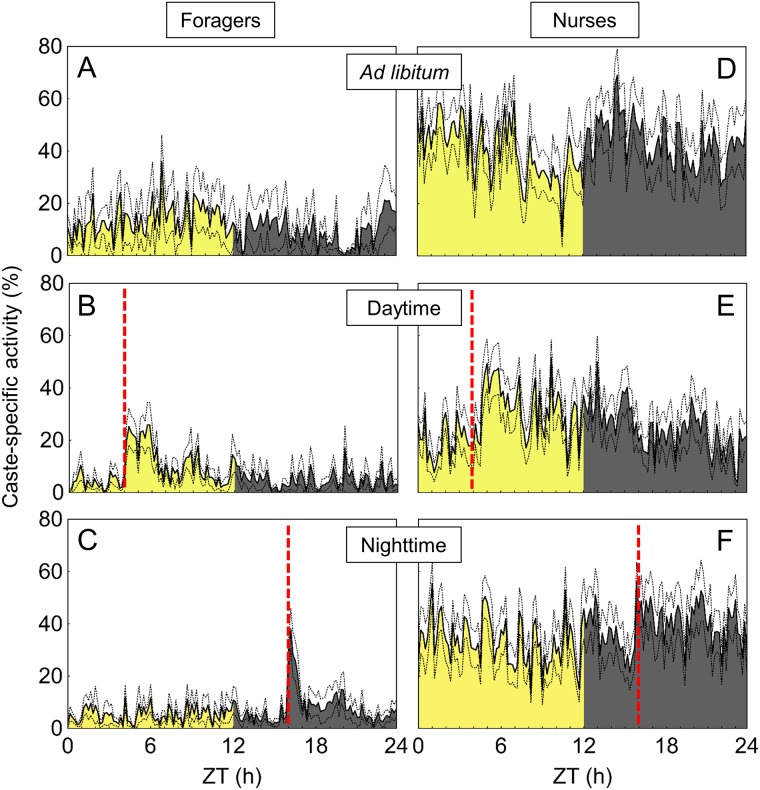
Effect of feeding regime on caste-specific activity patterns in the social context, for both foragers and nurses. Mean activity (solid lines) and mean±SE (dashed lines) are shown for the day phase (yellow area) and the night phase (black area) in relation to zeitgeber time (ZT). Feeding times (daytime feeding: ZT 4; nighttime feeding: ZT 16) are indicated as dashed red lines; (A): foragers (n = 8) under *ad libitum* feeding; (B): foragers (n = 12) under daytime feeding; (C): foragers (n = 12) under nighttime feeding; (D): nurses (n = 13) under *ad libitum* feeding; (E): nurses (n = 12) under daytime feeding; (F): nurses (n = 12) under nighttime feeding.

Average patterns of caste-specific activities, as described in Table 1, were calculated to evaluate at which daytimes foragers and nurses were active in the social context (Fig 6; see also Figure A in S1 File for examples of activity profiles of single foragers and nurses in the social context). Under *ad libitum* feeding of the subcolonies, foragers were similarly active during the light and dark phase (Fig 6A). Under the restricted feeding regimes, foragers displayed prominent peaks of activity coupled to the food availability, either at the light or the dark phase (Fig 6B and 6C, for further statistical analysis see Figure B in S1 File). In both cases, activity markedly increased after feeding and persisted high only for a short time thereafter. Independent of the feeding time, nurses were active both during the day and night time at constant high levels (Fig 6D–6F). Yet in both restricted feeding regimes, a trend of increased activity levels in the half-day period of actual feeding was observed, especially during nighttime feeding (see Figure B in S1 File).

**Fig 7:**
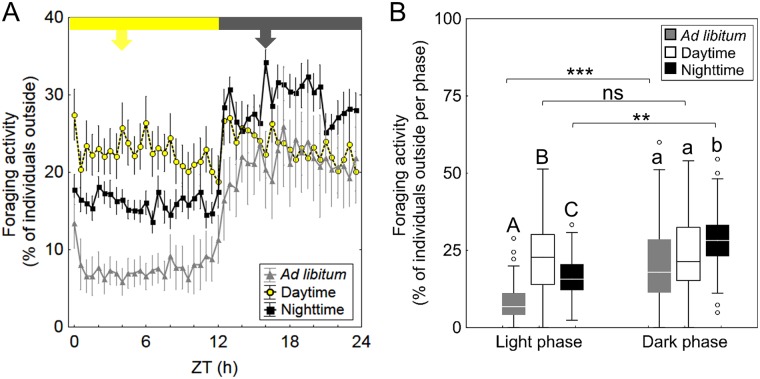
Effect of feeding regime on foraging activity in subcolonies. (A): Foraging activity in the course of 24 hours (mean±SE) for each feeding regime (grey: *ad libitum* feeding, n = 6 subcolonies; white: daytime feeding, n = 15 subcolonies; black: nighttime feeding, n = 12 subcolonies). (B): Foraging activity plotted separately for both the light and dark phases. Boxplots show medians (center lines) and interquartile ranges (boxes) for the three feeding regimes (grey: *ad libitum* feeding, n = 6 subcolonies; white: daytime feeding, n = 15 subcolonies; black: nighttime feeding, n = 12 subcolonies). Whiskers indicate the minimum and maximum values and open circles show outliers. Different capital letters show differences during the day phase and small letters show significant differences during the night phase between the feeding regimes (Kruskal-Wallis tests with Bonferroni correction, α = 0.017). Asterisks indicate differences between light phase and dark phase within every feeding regime (Wilcoxon signed-rank test with Bonferroni correction, α = 0.017). **: p<0.003; ***: p<0.0003; ns: p>0.017.

At the subcolony level, foraging activity was higher during the dark phase under both *ad libitum* and nighttime feeding (Fig 7, Wilcoxon signed-rank test with Bonferroni correction, α = 0.017; *ad libitum* feeding: T(288) = 157, z = 10.02, p<0.001; nighttime feeding: T(576) = 1033, z = 13.71, p = 0.001). Under daytime feeding, the daily activity pattern was not completely reversed, but activity levels in both phases changed, resulting in similar levels over the 24 h (Wilcoxon signed-rank test with Bonferroni correction, α = 0.017; T(720) = 21782, z = 1.29, p = 0.2). Thus, foraging activity during daytime feeding was significantly reduced in the dark phase and increased in the light phase (Kruskal-Wallis tests with Bonferroni-correction, α = 0.017; light phase: H(2, n = 792) = 248.68, p = 0.001; *ad libitum* vs. daytime: p<0.001 *ad libitum* vs. nighttime: p<0.001, daytime vs. nighttime: p<0.001; dark phase: H(792) = 69.63, p<0.001; *ad libitum* vs. daytime: p = 0.06, *ad libitum* vs. nighttime: p<0.001, daytime vs. nighttime: p = <0.001).

#### Daily locomotor activity of foragers and nurses in isolation from the social context

**Fig 8:**
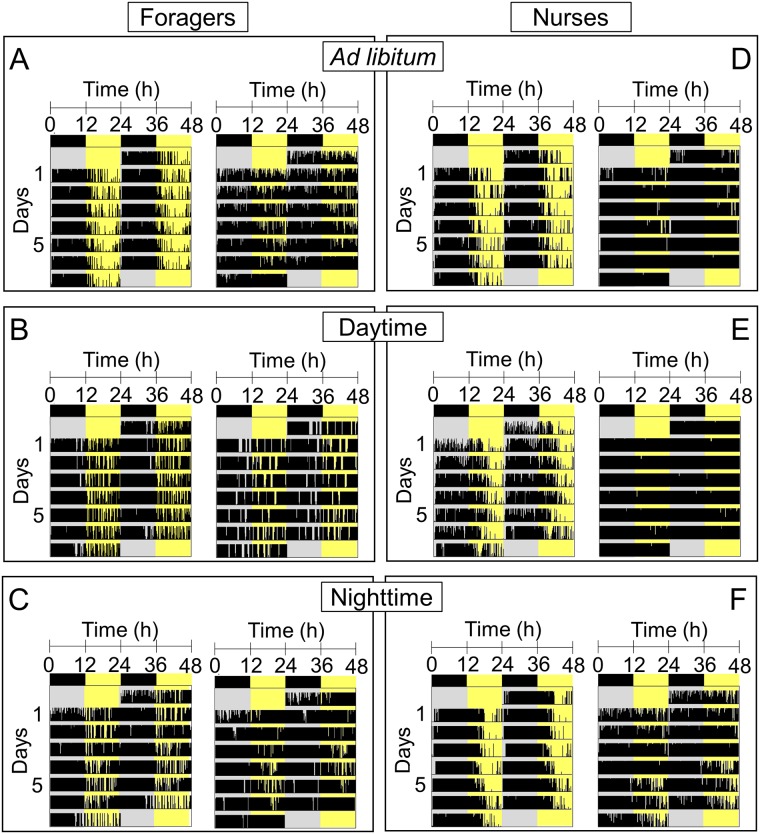
Examples of actograms of workers in isolation from the social context. Locomotor activity (indicated as black bars) is shown as double plot for single workers of the nurse and forager caste under a 12:12h LD-cycle. For the three feeding regimes, actograms of one rhythmic (left) and of one arrhythmic individual (right) are shown. (A): forager after *ad libitum* feeding of the subcolony; (B): forager after daytime feeding of the subcolony; (C): forager after nighttime feeding of the subcolony; (D): nurse after *ad libitum* feeding of the subcolony; (E): nurse after daytime feeding of the subcolony; (F): nurse after nighttime feeding of the subcolony.

Survival and rhythmicity of isolated workers strongly depended on caste affiliation (Table 3). Independent of the feeding regime, survival rate in nurses was roughly twice as high as in foragers. In both castes, rhythmic and arrhythmic individuals were present (Fig 8), but their proportions varied depending on caste and feeding regimes (Table 3). Approximately 70% of the isolated foragers were rhythmic under *ad libitum* feeding of the subcolonies. Under the restricted feeding regimes, 20 to 30% of the foragers showed circadian rhythms. Most of the nurses (80 to 90%), in turn, were rhythmic in isolation, independent of the feeding regime, yet displayed arrhythmicity in the social context as described above. For further comparisons of the activity patterns, see Figure C in S1 File.

**Table 3:** Effect of feeding regime on rates of survival and rhythmic daily activity of isolated individuals of both castes in locomotor activity monitors.

Feeding regime	Survival rate (%)			Rhythmicity (%)		
	Forager	Nurse	χ^2^ test	Forager	Nurse	χ^2^ test
*Ad libitum* feeding	34.6 (n = 9)	74.4 (n = 32)	** χ^2^ = 8.8	66.7 (n = 6)	90.6 (n = 29)	ns χ^2^ = 3.2
Daytime feeding	38.0 (n = 30)	66.7 (n = 88)	*** χ^2^ = 16.5	33.3 (n = 10)	76.1 (n = 67)	*** χ^2^ = 18.1
Nighttime feeding	41.1 (n = 23)	84.3 (n = 86)	*** χ^2^ = 31.6	17.4 (n = 4)	80.2 (n = 69)	*** χ^2^ = 32.4
χ^2^ test	ns χ^2^ = 0.2	* χ^2^ = 9.4		ns χ^2^ = 7.2	ns χ^2^ = 3.1	

Differences between castes within a feeding regime and differences within castes between feeding regimes via χ^2^ tests under Bonferroni correction.

*: p<0.017;

**: p<0.003;

***: p<0.0003;

ns: p>0.017).

## Discussion

### Locomotor rhythms of isolated *C*. *rufipes* workers

Based on the daily locomotor rhythms of isolated forager and nurse workers under a 12:12h LD-cycle, *C*. *rufipes* can be classified as a nocturnal species, thus complementing previous descriptions about the predominantly nocturnal foraging activity observed in both the laboratory and in the field [2, 25, 26]. As individuals immediately shifted their activity following a six hour delay of the light phase, they were truly entrained by the LD-cycle [55]. Light can be claimed as a strong zeitgeber for the endogenous clock, since period lengths of the workers’ locomotor activity strongly resembled the 24 hour period of the imposed light-dark cycle. The prominent activity peaks after the light transitions are most likely a result of stress reactions of the individuals to the step changes in illumination and likely represent a masking effect [55]. To clarify this assumption, activity could be monitored in the future under increasing/decreasing light intensities simulating twilight conditions. Under constant darkness, the locomotor activity rhythms of both castes started to run free from the entrained activity, and drifted with period lengths close to 24 hours, i.e. the rhythm represents the output of a circadian, endogenous clock.

As we found no differences in levels of nocturnal activity, rhythmicity and period lengths in LD- and DD-regimes, we demonstrate the presence of a functional endogenous clock in both castes. The presence of some arrhythmic individuals in both castes might not be ascribed to a missing endogenous clock in these ants, but rather to their stressful reactions because of the prolonged social isolation.

In contrast to our results, other studies reported strong differences in locomotor rhythms between castes in other ant species. In *Diacamma* ants, young workers, likely nurses, exhibited strong rhythmic activity, whereas older workers, likely to behave as foragers, did not [43]. The authors argued that this may reflect the arrhythmic foraging activity observed in the field. However, the illustrated arrhythmicity and increased activity levels in *Diacamma* foragers could also be the result of an aging clock, as previously shown in *Drosophila* [60, 61]. Conversely, major and media workers in *C*. *compressus*, likely foragers, were rhythmic, while minor workers, which likely perform inside-nest activities, showed no circadian rhythmicity [41]. The authors hypothesized that the lack of rhythmicity in in-nest workers might reflect their all-around-the-clock working schedule within the colony, but no behavioral correlates of activity in the social context were provided. This is why we aimed at monitoring behavioral activity of foragers and nurses of *C*. *rufipes* in the social context of the colony, and extended the analysis of their rhythmic activity in subsequent isolation.

### Caste-dependent plasticity of daily behavioral rhythms

As temporal changes in food availability may strongly influence the timing of activities, synchronization of daily activity patterns in both foragers and nurses was evaluated in subcolonies under restricted and *ad libitum* feeding regimes. For the first time in social insects, behavioral activities were continuously observed over 24 hours as ethograms for single nurses and foragers with the aid of detailed and enormously time-consuming manual tracking in long-term video recordings. One fundamental difference in activity profiles of the two castes was the variation in inactivity levels. Foragers remained inactive up to 50% of their time and always showed lower caste-specific activity levels than nurses, for all feeding regimes. This may in part result from the small size of the experimental setup, as foragers did not need to cover long distances to collect food as in the natural context. With 30% of their time, nurses showed considerable levels of inactivity as well. Such high inactivity levels were already described for workers of several ant species in the laboratory [30, 62–64] as well as in the field [65], and are therefore not a simple laboratory artifact. Even though ants in those studies were only observed for short time windows, our continuous observations confirm the presence of high inactivity levels over the whole day. Although inactivity levels of foragers were lower and similar to those observed in nurses under *ad libitum* feeding, they spent their active time with different behavioral activities. Foragers performed predominantly antennating and walking, behaviors classified as non-specific as they are performed by all workers. In a broader sense, however, these activities reflect the search for food in this caste. In addition, foragers showed always lower caste-specific activity levels than nurses because they synchronized their activity with food availability and the light-dark-cycle. Consequently, behavioral activity patterns of foragers were predominantly circadian. Only under nighttime feeding, activity levels in many foragers were markedly low and therefore only few circadian activity patterns could be detected under this feeding regime. Nurses were active all around the clock tending the brood, so mostly ultradian rhythmicities could be detected for their behavioral activity patterns.

Interestingly, although counts of foragers outside the nest under the *ad libitum* feeding regime indicated that the preferred foraging time at the group level occurred in the first hours of the night, continuous tracking of random individuals, however, showed similar foraging intensity in both phases of the day. Such flexibility may enable foragers to adjust their activity in correlation with temporal changes in food availability, an essential ability in ants that collect carbohydrate-rich food in renewable sources such as extrafloral nectaries or aphid colonies [2, 25]. In this respect, *C*. *rufipes* was reported to forage during day and nighttimes [2, 25–27] and field observations by one of the authors (FR) suggest that *C*. *rufipes* colonies may seasonally shift their activity from nocturnal to diurnal foraging, like many other ant species do [66, 67].

In contrast to foragers, nurses were active in both phases of the daily cycle with high activity levels, mostly at the brood pile, likely to meet the needs of the brood. Consequently, feeding and licking of the brood was the most dominant activity in nurses, taking up to 40% of their time. A study on brood care in *Solenopsis invicta* showed that larvae are fed up to 50 times per hour and are patrolled 200–800 times per hour, which could lead to uniform levels of nourishment for all larvae [68]. All-around-the-clock activity as observed in nurses appears to be a widely-occurring adaptive behavior in social insects, as it was also described for in-hive activities of honey bees [45], and recently detected in intra-nest activities in *Temnothorax* ants [69]. In our experiments, the feeding regimes had only negligible effects on the daily activity pattern of nurses. We observed a short increase of activity after feeding, which could be ascribed to stronger interactions with the returning foragers and suggests a form of social synchronization. Inter-caste interactions occurred predominantly via trophallaxis inside the brood chamber, where members of both castes spent comparable amounts of time. This emphasizes the temporal connection between the castes via food flow, as food is passed from returning foragers to nurses and afterwards to the brood. A study in *Leptothorax* ants already described such alteration of in-nest movement activity by returning foragers [70], analogous to the increase in caste-specific activities of nurses in our study.

As in honey bees, arrhythmicity at the colony level in *C*. *rufipes* was not based on rhythmic activity of individuals working in shifts or out of phase [46], but on the arrhythmic activities of single inside-nest workers (see S1 File). A shift system was proposed for ants because of spontaneous changes in period lengths and phase-shifts of free-running locomotor rhythms in isolated *C*. *compressus* workers [40, 41]. Based on our detailed study of in-nest activities, the existence of shifts to explain arrhythmicity can be ruled out in *C*. *rufipes*, as recently done in *Temnothorax rugatulus* ants as well [69].

### Comparison between behavioral rhythms in the social context and locomotor activity rhythms in isolation

After having spent 14 days in the social context, individual foragers and nurses were monitored in isolation, thus allowing the comparison of daily behavioral activities with endogenous locomotor activity rhythms in the same workers. Irrespective of the preceding feeding regime, most nurses showed rhythmicity and night activity. This result again demonstrates that nurses possess a functional endogenous clock and are able to entrain to the LD-cycle, although they are behaviorally arrhythmic under this zeitgeber in the social context. Therefore, the output of the endogenous clock must be either masked or decoupled in the social context [51]. As another output of their clock, *C*. *rufipes* nurses show circadian changes in temperature sensitivity for brood care [34], and display a daily rhythm in brood translocation between different temperatures [71]. Hence, some nursing behaviors seem to be performed in a rhythmic manner whilst others not. Since our experiments were done under constant temperature conditions, rhythmic brood translocation could not be observed. Future experiments are needed to clarify whether the same workers exhibit both rhythmic and arrhythmic components of nursing behavior and most notably, which zeitgebers synchronize rhythmic brood care inside the dark nest. Promising candidates are non-photic zeitgebers like cyclic social interaction [53, 72, 73], as well as daily cycles in temperature and humidity.

Only after *ad libitum* feeding of the subcolonies, isolated foragers showed similar levels of rhythmicity and night activity like nurses, confirming the results obtained in Experiment 1. After the temporally restricted feeding regimes, the majority of foragers in contrast displayed arrhythmicity. This finding was unexpected because the same workers showed rhythmic foraging activity in the social context. This suggests that foragers may have experienced food shortages or suffered stress during the short feeding time-windows, as a result of the observed drastic increase of arousal and activity. The additional highly reduced survival of foragers indicates that they could be less robust than nurses under isolation and may have lost their rhythmicity. This effect could be a consequence of aging, since foragers are usually older than nurses [74] and age negatively affects survival under social isolation in ants [75]. Thus, not only caste affiliation but also previous rearing conditions significantly affect individual activity still in isolation. Further studies on ants of known age and caste affiliation are needed to analyze the performance of an aging clock in combination with an age-dependent polyethism. Although only the minority of foragers was rhythmic after the restricted feeding regimes, these workers were uniformly night active. Despite their previous synchronizing with a specific time point in the night or even their switch to day activity in the social context, they still displayed their endogenous rhythms in isolation and did not show any long-term effects of the previous entrainment.

Our study indicates that ants show flexible daily behavioral rhythms that are context-dependent in multiple ways. Endogenous daily activity patterns of individuals belonging to specific castes are modified in the social context based on specific task demands. Such a mechanism was previously described only in the honey bee [52], but ants seem to share the link between division of labor and socially-mediated plasticity in activity rhythms. In honey bees, too, all workers possess a circadian clock and show rhythmicity in isolation of the social context [51]. Yet this endogenous rhythmicity is not observed in honey bee nurses in the social context [45, 46]. Therefore, temporal organization in *C*. *rufipes* ants and honey bees appear to share similar basic features. In this regard, the molecular characterization of an ant clock in the species *Solenopsis invicta* showed that both bees and ants own a mammalian-like clock with similar mechanisms [76]. Expression patterns of clock genes hereby reflect task specific activity patterns and link division of labor with clock function in bees [77] as well as in ants [42]. Although their endogenous clock awaits molecular characterization, ants of the genus *Camponotus* appear to be a promising model system to further explore the link between chronobiology and sociobiology.

## Supporting Information

S1 File Supplementary figures. (PDF)

S1 Table Complete raw data. (XLSX)
