# Supplementary material for: Plasticity of Daily Behavioral Rhythms in Foragers and Nurses of the Ant Camponotus rufipes: Influence of Social Context and Feeding Times
Source: PLoS One. 2017 Jan 18;12(1):e0169244. doi: 10.1371/journal.pone.0169244 (PMC5242425; doi:10.1371/journal.pone.0169244)
Supplement: S1 File — (PDF) [file pone.0169244.s001.pdf]

Plasticity of Daily Behavioral Rhythms in Foragers and Nurses of the Ant *Camponotus rufipes* – Supplementary figures

Activity profiles of foragers and nurses in both the social context and isolation

In Fig A we show examples of activity profiles of single foragers and nurses in both the social context and in isolation, for the three assayed feeding regimes. In the social context, levels of total activity in foragers were lower than in nurses and showed no daily pattern (Fig A column 1). As forager and nurses executed different behavioral activities, we calculated caste-specific activity profiles (Fig A column 2) to determine differences in the temporal organization of foragers and nurses. Although locomotor activity of foragers in the social context rather reflected their caste-specific activity (compare columns 2 and 3), nurses showed hardly any locomotor activity in the social context (Fig A column 3). Although mostly stationary, nurses were highly active displaying nursing behaviors. Thus, the locomotor activity profile failed to represent the characteristic, high level activity pattern of nurses.

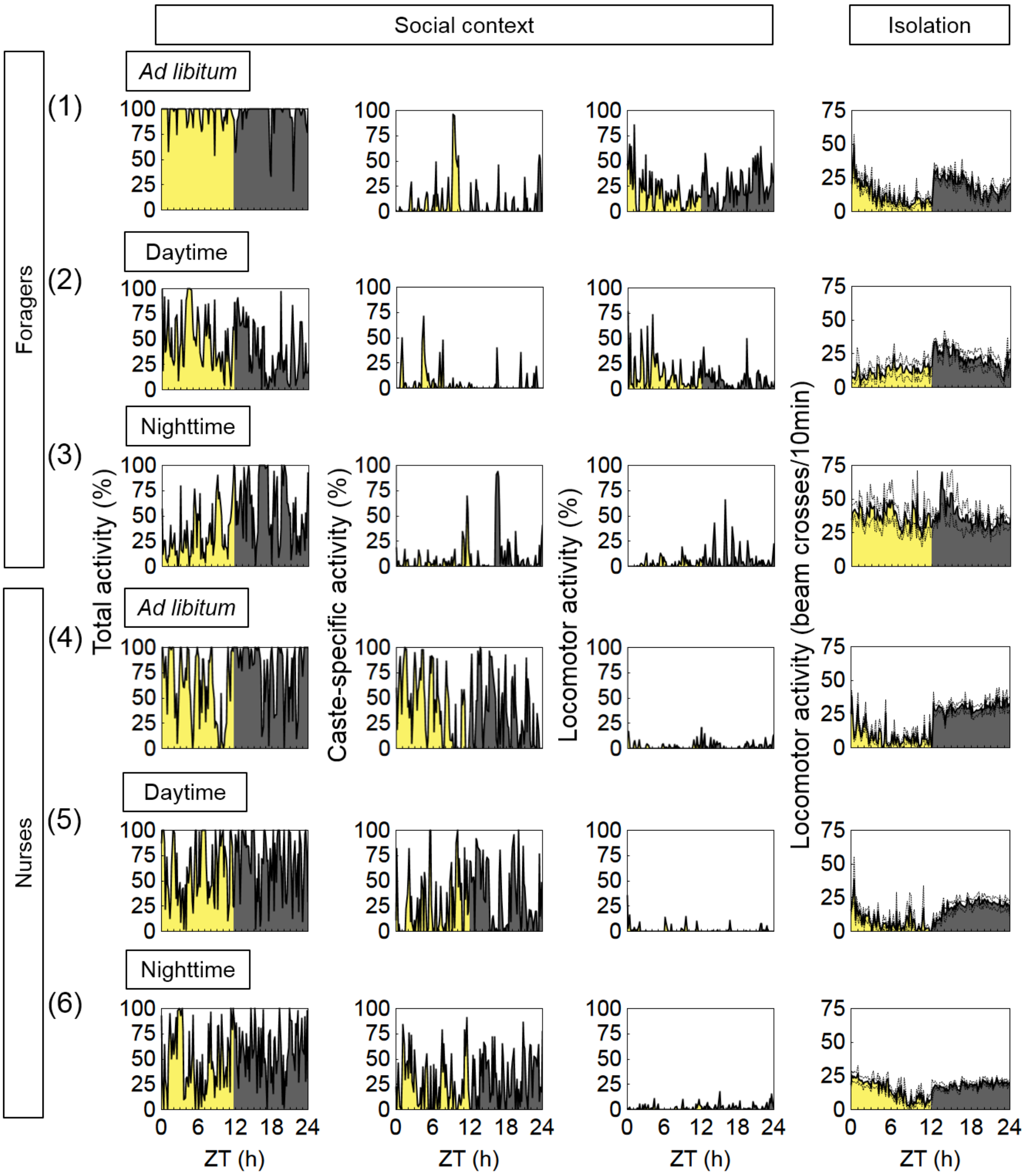

Fig A: Examples of activity profiles of foragers and nurses in both the social context and isolation, for the three assayed feeding regimes. (1): Forager under *ad libitum* feeding. (2): Forager under daytime feeding. (3): Forager under nighttime feeding. (4): Nurse under *ad libitum* feeding. (5): Nurse under daytime feeding. (6): Nurse under nighttime feeding. Column 1: Total activity levels in the social context. Column 2: Caste-specific activity levels in the social context. Column 3: Locomotor activity in the social context. Column 4: Locomotor activity in isolation. Caste-specific activities of foragers in these examples are circadian (*ad libitum*: τ= 20.0, daytime: τ=20.0h nighttime: τ=22.1h), and those of nurses are infradian (*ad libitum*: τ=12.9h, daytime: τ=7.4h, nighttime: τ=1.6h).

Experiment 2: Quantitative analysis of behavioral activity patterns in the social context

Relative levels of caste-specific activity during the night phase were determined by normalizing data sets of every individual for total activity of each ant. This method provides comparison despite high inter-individual and inter-caste variances in activity levels. To determine day or night activity, levels of nocturnal caste-specific activity were tested against random activity in both the light and the dark phase for the two castes and the three assayed feeding regimes (one-sample t-test against 50% with Bonferroni correction, α=0.025). Within feeding regimes, we compared levels in nocturnal activity between castes (unpaired t-tests under Bonferroni-correction, α=0.025).

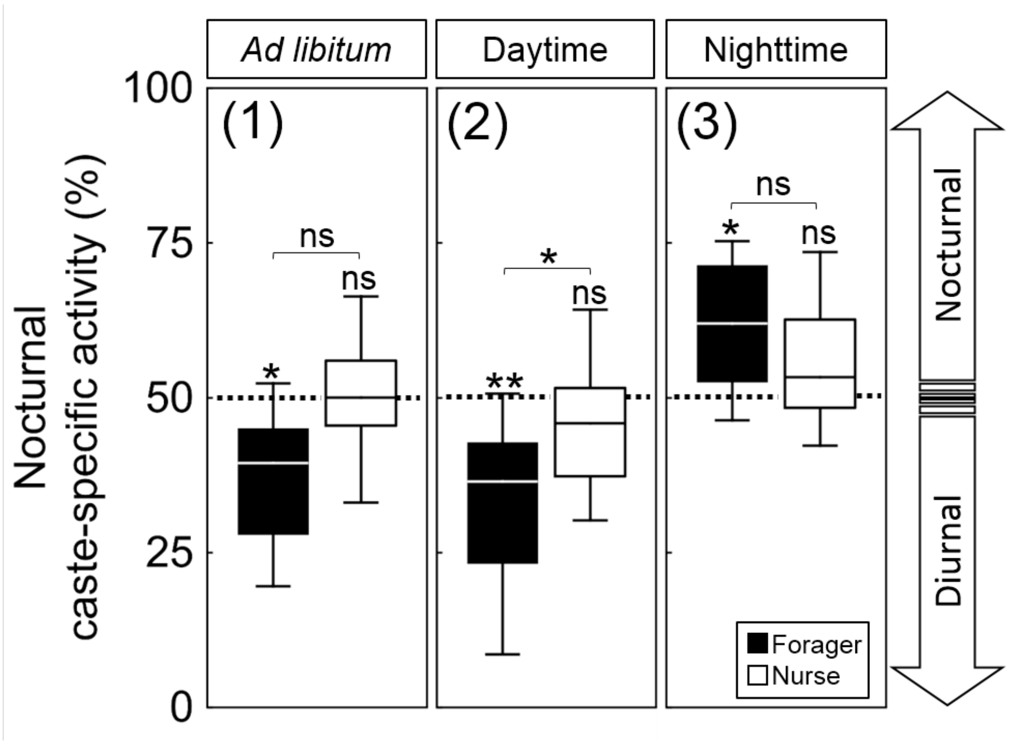

Fig B: Effect of feeding regime on nocturnal caste-specific activity of both foragers and nurses. Boxplots show medians (center lines) and interquartile ranges (boxes) for foragers (black) and nurses (white), for the three feeding regimes. (1): *ad libitum* feeding; (2): daytime feeding; (3): nighttime feeding. Whiskers indicate the minimum and maximum values. Asterisks show significant differences from random distribution for every caste and feeding regime (one sample t-test against 50% with Bonferroni correction, α=0.025) and significant differences between castes for every feeding regime (unpaired t-test with Bonferroni correction, α=0.025). ns: p>0.025, *: p<0.025, **: p<0.005).

Experiment 3: Quantitative analysis of locomotor activity patterns in isolation

Average activity patterns of foragers and nurses were calculated separately for the rhythmic and arrhythmic individuals, and the most common pattern was used for further comparisons. The largest proportion of individuals was rhythmic under all three feeding regimes in nurses, and under *ad libitum* feeding in foragers. High proportions of arrythmic individuals were observed only under restricted feeding in foragers. Rhythmic foragers under *ad libitum* feeding (Fig C (1)) and rhythmic nurses under all feeding regimes (Fig C (4)-(6)) were mostly nocturnal. In contrast, the representative activity profiles of arrhythmic foragers after the restricted feeding (Fig C (2)-(3)) depicted a clear reduction in night activity as well as damped overall activity levels compared to the other activity patterns.

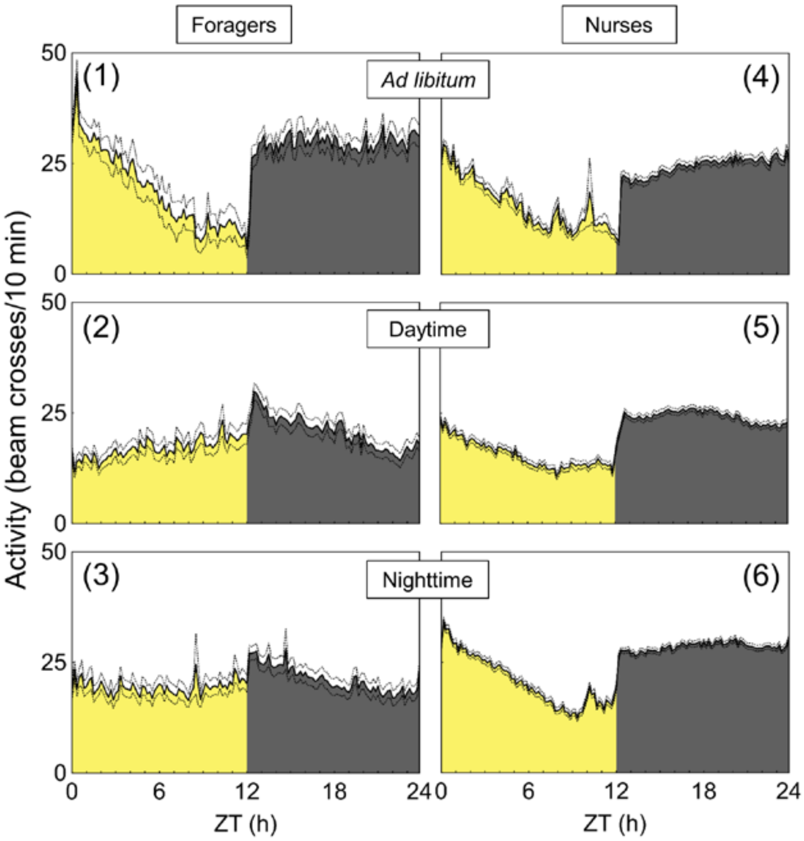

Fig C: Effect of feeding regime on diel locomotor activity of isolated nurses and foragers. Average activity (mean: solid lines; mean±SE: dashed lines; day phase: yellow area, ZT 0-12; night phase: grey area, ZT12-24). (1): foragers (n=6) after *ad libitum* feeding of the subcolony; (2): foragers (n=20) after daytime feeding of the subcolony; (3): foragers (n=19) after nighttime feeding of the subcolony; (4): nurses (n=29) after *ad libitum* feeding of the subcolony; (5): nurses (n=67) after daytime feeding of the subcolony; (6): nurses (n=69) after nighttime feeding of the subcolony.
